# Evaluation of the Effect of Fixation Angle between Polyaxial Pedicle Screw Head and Rod on the Failure of Screw-Rod Connection

**DOI:** 10.1155/2015/150649

**Published:** 2015-02-22

**Authors:** Engin Çetin, Mustafa Özkaya, Ümit Özgür Güler, Emre Acaroğlu, Teyfik Demir

**Affiliations:** ^1^Department of Orthopaedics, International Hospital, Yeşilköy, 34149 İstanbul, Turkey; ^2^Department of Mechanical Engineering, TOBB University of Economics and Technology, 06560 Ankara, Turkey; ^3^Ankara Spine Center, Kavaklıdere, 06700 Ankara, Turkey

## Abstract

*Introduction.* Polyaxial screws had been only tested according to the ASTM standards (when they were perpendicularly positioned to the rod). In this study, effects of the pedicle screws angled fixation to the rod on the mechanical properties of fixation were investigated. *Materials and Method.* 30 vertically fixed screws and 30 screws fixed with angle were used in the study. Screws were used in three different diameters which were 6.5 mm, 7.0 mm, and 7.5 mm, in equal numbers. Axial pull-out and flexion moment tests were performed. Test results compared with each other using appropriate statistical methods. * Results.* In pull-out test, vertically fixed screws, in 6.5 mm and 7.0 mm diameter, had significantly higher maximum load values than angled fixed screws with the same diameters (*P* < 0.01). Additionally, vertically fixed screws, in all diameters, had significantly greater stiffness according to corresponding size fixed with angle (*P* < 0.005). * Conclusion.* Fixing the pedicle screw to the rod with angle significantly decreased the pull-out stiffness in all diameters. Similarly, pedicle screw instrumentation fixed with angle decreased the minimum sagittal angle between the rod and the screw in all diameters for flexion moment test but the differences were not significant.

## 1. Introduction

Pedicle screw-rod systems have almost been the standard method of fixation in the treatment of various spinal disorders following advancement by Cotrel and Dubousset. Studies reported that use of pedicle screw fixation allowed surgeons to apply enhanced corrective forces to spine and advantages of the pedicle screw fixation compared with hook or hybrid systems [1–4]. Ascending use of pedicle screw fixation as a treatment method in incidence of adult deformity correction procedures, particularly osteotomies and long fusions to pelvis, poor bone quality of aging populations, and increased biomechanical stresses on fixation materials still causes the problems despite of new developments in spinal instrumentation field. Studies revealed that problems such as pedicle and vertebral body fractures, instrumentation failures, pseudarthrosis, and adjacent segment degenerations occurred after pedicle screw instrumentation [5–11]. Besides those problems, some complications like cerebrospinal fluid leak, deep infection, and nerve root injury were also reported [10, 12].

Stability of the pedicle screw constructs have been evaluated mainly by pullout, insertional torque, and cyclic loading tests [13]. Numerous studies have been performed to demonstrate the factors affecting the ability and stability of the pedicle screw fixation. Vertebral body bone mineral density is an important factor in the stability of the pedicle screw fixation [13–16]. Anatomy of vertebral pedicles is another important factor because of pullout strength capacity of the pedicle [17, 18]. Pedicle screw design, outer and inner diameters of the screw, and screw insertion techniques are other main factors affecting the stability of the pedicle screw fixation [19–31].

Understanding the degree of freedom's importance on the pedicle screw head was a significant milestone in development of the pedicle screw fixation. Monoaxial pedicle screws were firstly used in spinal fixation and provided effective correction for the deformity in coronal, sagittal, and axial planes. However, difficulties in achieving lodgement of the rod into the screw head for monoaxial pedicle screws in regions such as lumbosacral and sacropelvic and arrangement in the insertion depth of the screw compelled the surgeons during the operations. Additionally, following the operation, the increased stress could occur in interface of instrumentation and fixed segments, and loss of fixation could also occur. To overcome the disadvantages of the monoaxial screws, polyaxial pedicle screws were then used. Polyaxial pedicle screw fixation eased the achieving appropriate contact between the instrumentation and fixed segments in the surgical operations due to its degree of freedom capacity. There were many studies which compare the biomechanical effects of the monoaxial and polyaxial pedicle screw fixations [32–36]. In those studies, it was concluded that the loads in the interface of the bone and implant could be reduced with increasing the degree of freedom of the implant. It was also said that fixation-bone failures could be prevented with use of polyaxial screws. Fogel et al. [36] showed that the polyaxial head coupling to the screw was the first part which fails. From this reason it may provide protection for pedicle screw and prevent pedicle screw breakage.

**Figure 1:**
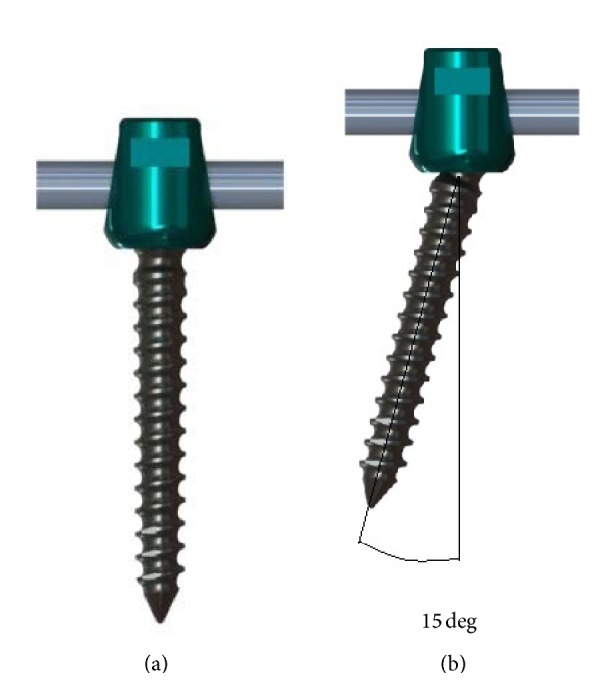
(a) Vertically fixed and (b) fixed with angle.

Although polyaxial screw was commonly used due to its advantage of easy rod connection, the screws are tested only in the perpendicular position (Figure 1(a)) according to American Society for Testing and Materials (ASTM F1798) standards for testing screws. It should be taken into account that the screws are also used with angled position (Figure 1(b)) during surgical operations.

Stiffness can be described as the rigidity of a structure. It can be calculated by the load versus displacement curve. The slope of the linear elastic portion of load versus displacement curve gives the stiffness.

This study was raised from the senior authors' observations, polyaxial screw failures (head dislodgement, cap loosening, and screw pullout) mostly during or following deformity correction procedures. In the present study it is hypothesized that angled fixed pedicle screws to the rods, as similar as surgical applications, have lower stiffness. Screws with larger core diameters may be of disadvantage in this respect. The purpose of this study is to assess the effect of pedicle screw head-shaft fixation angles on the mechanical properties of pedicle screws.

## 2. Experimental Procedure

Axial pullout test and flexion moment test were performed to investigate the polyaxial pedicle screw failure in the present study. Sixty titanium polyaxial pedicle screws (Osimplant, Turkey), with 45 mm length, were used. The screws with three different outer diameters, 6.5 mm, 7.0 mm, and 7.5 mm, were used and each group had 20 screws.

**Figure 2:**
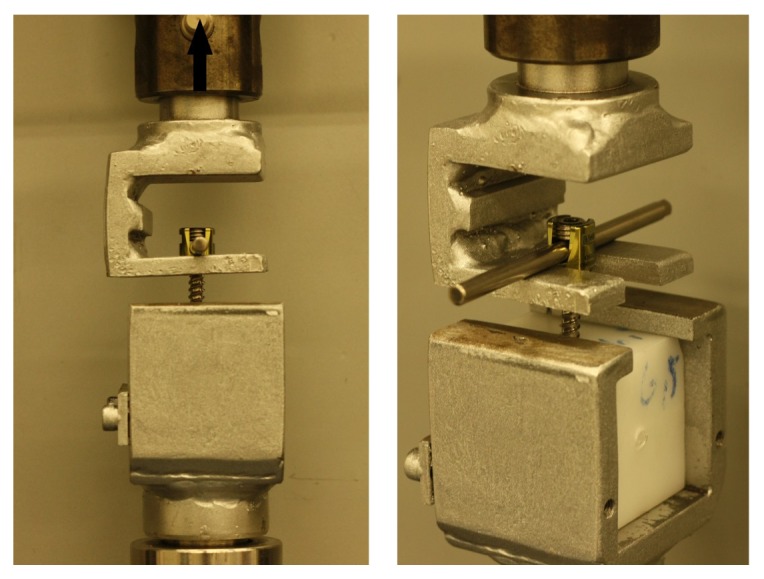
Axial pullout test setup.

Screws, in equal numbers, were fully inserted perpendicularly into ultrahigh molecular weight polyethylene (UHMWPE) blocks and fixed to 6.0 mm titanium rods with 0 or 15 degrees of screw head/shaft angles on sagittal plane as can be seen in Figure 1. All screw nuts were inserted to screw head with the torque of 10 N·m. Thirty screws were sustained to axial pullout test. Polyethylene blocks with inserted screws and connected rods were mounted into a metal polyethylene holding jaw and perpendicular pullout force was applied to rods with Instron 3300 Testing Frame as can be seen in Figure 2. Polyethylene holding jaw was angled 15 degrees during the examination of angled fixed screws so that a perpendicular pullout force could be obtained. Tensile load was applied to the test material with 5 mm/min crosshead speed until the screw head dislodges from the screw shaft. Maximum load (N) (the load at which the screw head dislodges from the screw shaft), displacement at maximum load (mm), and stiffness (N/mm) values were recorded for each screw.

**Figure 3:**
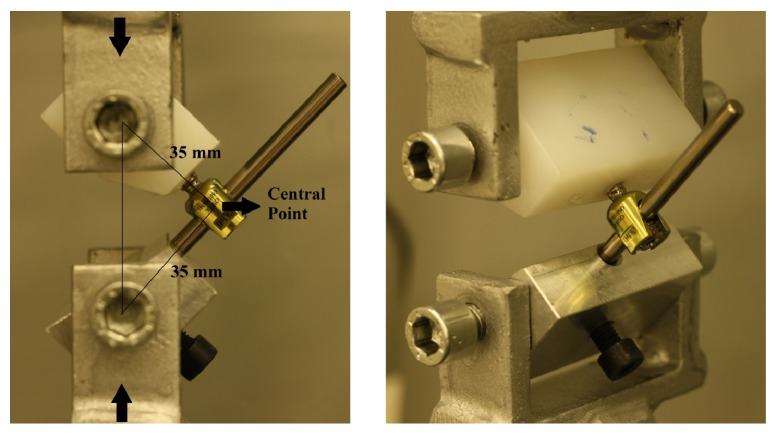
Flexion moment test setup and central point.

Remaining screws were sustained to flexion moment test. The test frame and crosshead speed were the same with the axial pullout test. Polyethylene blocks holding the screws and a metal block holding the rods were fixed into apparatuses allowing the flexion and downward vertical force was applied to system until the screw head cut-off. The test setup for flexion test can be seen on Figure 3. The central point of the screw head-rod system was located 35 mm away from the rotation centres [37] and it was also shown in Figure 3. Maximum load and displacement at maximum load values were recorded for each screw by testing software. With the help of trigonometric calculations, moment values at maximum load (N·mm) were calculated for each screw. Minimum sagittal angle values, referring the minimum angle between the rod and the screw at the maximum load, were also calculated.

Study groups were formed by screw diameter (6.5, 7.0, and 7.5 mm), fixation angle of screw head to rod (0 degree [vertical]/15 degrees [angled]), and the applied test (pullout and flexion moment); thus 12 groups, each group containing 5 samples, were obtained. Data were statistically analysed using Microsoft Excel 2010; two-tailed Student's *t*-tests were performed to compare the results for different groups.

## 3. Experimental Results

### 3.1. Axial Pullout Testing

**Figure 4:**
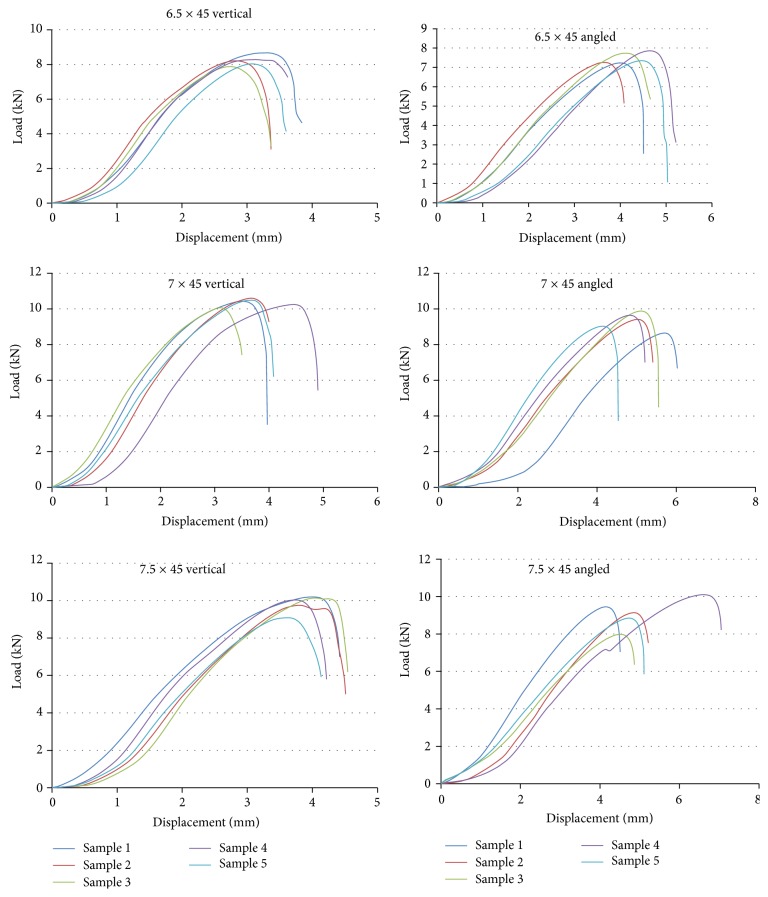
Load versus displacement curves of the vertical and angled fixations.

7.0 mm vertically fixed screws had the highest maximum load values (pull-out strength) followed by 7.5 mm and 6.5 mm vertically fixed screws. 7.0 mm vertically fixed screws had also the highest stiffness values followed by 6.5 mm and 7.5 mm vertically fixed screws as can be seen on Table 1. The load-displacement curves of the vertical and angled fixations for axial pullout test are shown in Figure 4. As can be seen on load versus displacement curves, stiffness of the fixations is calculated by the linear elastic portion of the curves. Vertically fixed screws, in 6.5 mm and 7.0 mm diameter, had significantly higher maximum load values according to corresponding size fixed with angle (*P* < 0.01) as given in Table 2. 7.0 mm vertically fixed screws had significantly higher maximum load than 6.5 mm vertically fixed screws (*P* < 0.0001). Similarly, 7.5 mm screws had significantly higher maximum load than 6.5 mm screws with the *P* value of 0.0003 when they fixed vertically. 6.5 mm screws fixed with angle had significantly lower maximum load than 7.0 mm screws fixed with angle (*P* = 0.0002) and 7.5 mm screws fixed with angle (*P* = 0.0074). There were no other significant differences for maximum load. Vertically fixed screws, in all diameters, had significantly greater stiffness according to corresponding size fixed with angle (*P* < 0.005). Although 7.5 mm vertically fixed screws had higher pullout strength than 6.5 mm vertically fixed screws; the latter was stiffer (*P* = 0.0498); others showed no significant stiffness difference according to the screw diameter.

### 3.2. Flexion Moment Testing

7.0 mm pedicle screws fixed with angle had the highest moment at maximum load and the lowest minimum sagittal angle values as can be seen in Table 3. However, statistical evaluation revealed that those values were not significantly different whether the screws were fixed vertically or with angle, and it is also valid for all corresponding diameters (Table 2). In the comparison of different diameters, 7.0 mm and 7.5 mm screws had significantly higher moment values than 6.5 mm screws either they were fixed vertically or with angle (*P* < 0.02). 6.5 mm screws had greater minimum sagittal angle values than 7.5 mm screws when they were fixed vertically (*P* = 0.048). 6.5 mm screws had greater minimum sagittal angle values than 7.0 mm screws when they were fixed with angle (*P* = 0.006).

## 4. Discussion

Many studies have been conducted about the factors which affect the stability of pedicle screws. Vertebral bone mineral density, anatomy of the pedicles and insertion techniques, and pedicle screw characteristics are directly related to pullout and fatigue strength [13–18, 25]. Outer diameter is the most important feature of the screw controlling pullout strength. As the outer diameter increases, the pull-out strength increases unless a cortical pedicle is purchased. Pedicle screw design, cylindrical or conical, outer or inner diameter configuration, and thread shape have been shown as the factors which affect the stability [19–31].

Polyaxial screw's mobile head design facilitates easier rod connection and provides solution for screw placement problems. Studies demonstrate that polyaxial screws are as effective as monoaxial screws in the coronal and sagittal plane correction but the latter provides greater correction of rotational deformity [32, 34]. Although polyaxial screws have been commonly used in daily practice conveniently to their purpose, screw heads are fixed to rods in angles and ASTM standards recommend testing them only in the perpendicular position.

**Figure 5:**
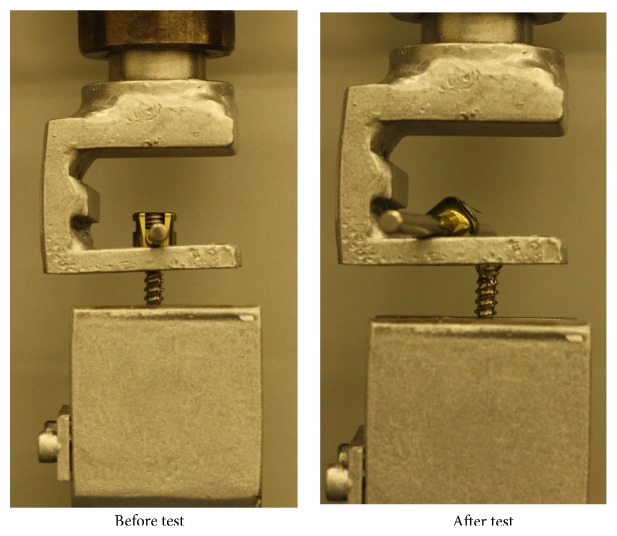
Pullout test, before and after.

In pullout test, all screw heads were pulled out from the screw in both vertically fixed and angled groups which can be seen in Figure 5. The results of present study clearly showed that pullout stiffness of polyaxial screws was decreased when they were fixed to rods with a head and shaft angle. Varying screw diameters did not change those results for 6.5, 7.0, and 7.5 mm screws. However, angled fixation of screws had no significant effect on the moment at maximum load and minimum sagittal angle values in flexion moment test according to ones fixed vertically. Angled fixation of screws reduced the pullout strength significantly for 6.5 mm and 7.0 mm screws.

There are not any other studies in the literature evaluating stability of polyaxial screws in this manner. Although polyaxial screws have been found to have more compression and flexion stiffness than the monoaxial screws with an anterior cage support at lumbosacral spine [33] and instrumentations with polyaxial screws have been shown to have lesser bone screw load level according to monoaxial screws [35], those studies compared polyaxial screws with monoaxial ones and emphasized the importance of proper screw rod settling in stability. There is not any existing study evaluating the effects of angular fixation on stability of polyaxial screws.

**Figure 6:**
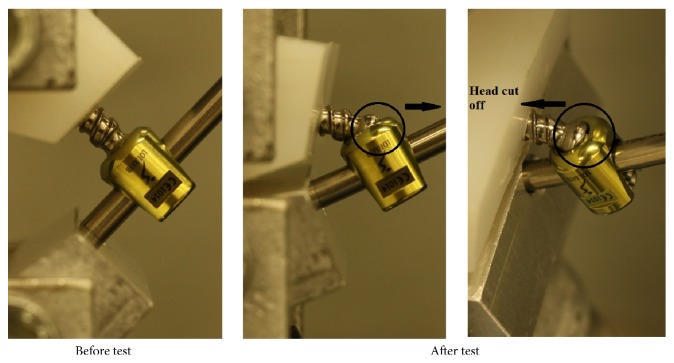
Flexion moment test, before and after.

**Figure 7:**
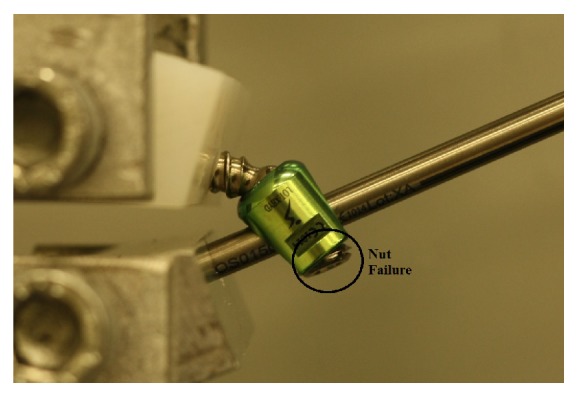
Nut failure before screw cut-off.

**Figure 8:**
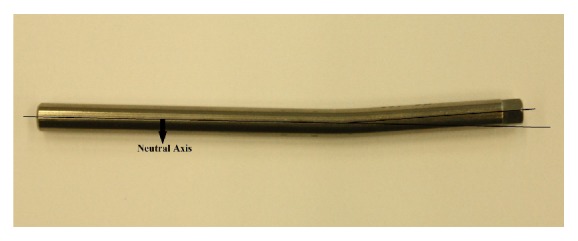
Rod failure in flexion moment test.

**Figure 9:**
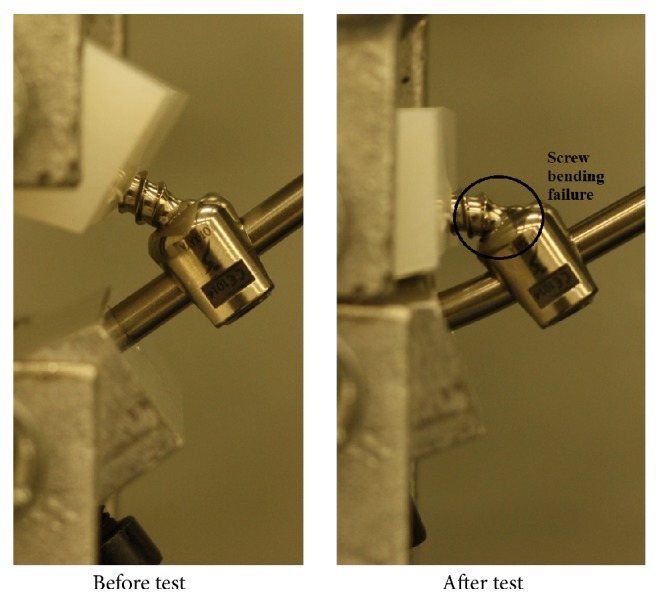
Screw bending failure in flexion moment test, before and after.

Polyaxial screw head coupling the shaft has been shown as the first part failing against load and this was suggested as a protective feature of the screw preventing screw or rod failure [36]. It was observed from the present study that all screw heads cut off from the screw in both vertically fixed and angled groups as can be seen in Figure 6 except 4 screws in the flexion moment test. For those 4 screws, the nuts failed before the screw heads were cut-off. The screws in which the nuts failed are shown in Figure 7. Rod failure in some samples was also observed as shown in Figure 8. There were also screw bending failures for 2 samples fixed with angle in flexion moment test as can be seen in Figure 9.

Increasing diameters are positively correlated with pullout strength and moment at maximum load values of the screws [23]. In the current study, 6.5 mm screws exhibited lower pullout strength than 7.0 and 7.5 mm screws when they were fixed vertically or angled statistically. Besides 7.0 and 7.5 mm screws had higher moment values than 6.5 mm either vertical or angled fixations. However, statistically only 6.5 mm vertically fixed screws were found to have greater stiffness than 7.5 mm screws, due to lesser displacement at maximum load of 6.5 mm. There were no observed differences between the 7.0 mm and 7.5 mm screws either they were fixed vertically or angled.

Minimum sagittal angle of 6.5 mm screws was higher than 7.5 mm when they fixed vertically and higher than 7.0 mm screws when they fixed with angle. Fixing the screw with angle decreased the minimum sagittal angle in all diameters but the differences were not statistically significant.

## 5. Conclusions

In the present study, the effects of angled fixation of pedicle screw to the rod on the mechanical properties of fixation were investigated. Fixing the pedicle screw to the rod with angle significantly decreased the pullout stiffness in all diameters for axial pullout test. Similarly, pedicle screw instrumentation fixed with angle decreased the minimum sagittal angle between the rod and the screw in all diameters for flexion moment test, but the differences were not significant.

## Figures and Tables

**Table 1:** Axial pullout test results.

	6.5 × 45		7.0 × 45		7.5 × 45	
	V	A	V	A	V	A
Maximum load (N)	8216	7485	10360	9320	9838	9102
Std.	300	289	210	491	457	780

Displacement at maximum load (mm)	3.02	4.18	3.67	4.96	3.84	4.98
Std.	0.23	0.39	0.49	0.56	0.17	0.96

Stiffness (N/mm)	2732.68	1800.87	2854.4	1899.48	2562.69	1863.99
Std.	141.26	137.86	344.67	241.24	64.05	271.30

**Table 2:** Statistical evaluation of test results.

	Maximum load	Pullout stiffness	Moment at maximum load	Minimum sagittal angle
6.5-V versus 6.5-A	0.0044^*^	0.0000^*^	0.5995	0.0655
7.0-V versus 7.0-A	0.0071^*^	0.0014^*^	0.1737	0.1407
7.5-V versus 7.5-A	0.1189	0.0050^*^	0.6761	0.0667
6.5-V versus 7.0-V	0.0000^*^	0.4978	0.0160^*^	0.0889
7.0-V versus 7.5-V	0.0607	0.1363	0.8019	0.5525
6.5-V versus 7.5-V	0.0003^*^	0.0498^*^	0.0129^*^	0.0479^*^
6.5-A versus 7.0-A	0.0002^*^	0.4577	0.0006^*^	0.0056^*^
7.0-A versus 7.5-A	0.6069	0.8324	0.6256	0.0507
6.5-A versus 7.5-A	0.0074^*^	0.6592	0.0176^*^	0.0620

^*^Statistical difference.

**Table 3:** Flexion moment test results.

	6.5 × 45		7.0 × 45		7.5 × 45	
	V	A	V	A	V	A
Maximum load (N)	843	796	1146	1283	1189	1234
Std.	103	45	162	141	190	252

Displacement at maximum load (mm)	20.14	13.02	25.33	19.37	23.64	16.28
Std.	1.81	2.78	5.26	2.57	2.70	1.23

Moment at maximum load (N·mm)	26791	25812	37653	43073	38728	40828
Std.	3598	1523	6364	4851	6743	8474

Minimum sagittal angle (°)	49.61	43.87	40.50	32.85	43.40	38.14
Std.	3.25	4.90	9.10	4.37	4.80	2.13
